# Effects of neratinib on angiogenesis and the early stage of the embryo using chicken embryo as a model

**DOI:** 10.17305/bb.2023.9869

**Published:** 2024-06-01

**Authors:** Hadeel Kheraldine, Arij Fouzat Hassan, Hashim Alhussain, Hamda Al-Thawadi, Semir Vranic, Ala-Eddin Al Moustafa

**Affiliations:** 1 College of Medicine, QU Health, Qatar University, Doha, Qatar; 2 College of Pharmacy, QU Health, Qatar University, Doha, Qatar; 3 Biomedical Research Center, Qatar University, Doha, Qatar; 4 Oncology Department, Faculty of Medicine, McGill University, Montreal, QC, Canada

**Keywords:** Neratinib, angiogenesis, embryogenesis, chorioallantoic membrane (CAM), tyrosine kinase inhibitor (TKI)

## Abstract

Angiogenesis is the process of forming new blood capillaries from pre-existing vessels. Even though it is essential during normal development, it plays a major role in cancer progression. Neratinib is a pan-human epidermal growth factor receptor (HER) inhibitor that has recently been approved for the treatment of HER2-positive breast cancer. However, its effects on angiogenesis and embryogenesis remain unknown. This study examined the antiangiogenic effects of neratinib using the chorioallantoic membrane (CAM) of chicken embryos. We also evaluated neratinib’s toxicity during the early stages of normal development using the chicken embryos, primary embryonic fibroblasts (EFBs), and human umbilical vein endothelial cells (HUVEC). Our findings revealed that neratinib significantly inhibited the CAM angiogenesis compared to controls by reducing vessel percentage area and the average vessel length. Furthermore, neratinib downregulated vascular endothelial growth factor (VEGF), a key mediator of angiogenesis. At lower concentrations, neratinib was well-tolerated during early stages of normal development. Additionally, EFBs treated with neratinib showed no morphological or viability changes when compared to controls. However, at the highest concentration tested, neratinib treatment reduced HUVEC cell viability. This effect may be associated with the dysregulation of key apoptotic genes, including caspase-3, caspase-8, caspase-9, and the B-cell lymphoma 2 (*Bcl2*) gene. Our findings indicate a novel potential application of neratinib as an antiangiogenic agent, exhibiting tolerable toxicity in the early stages of embryogenesis.

## Introduction

Angiogenesis is implicated in the pathogenesis of various diseases, including but not limited to cancer, arthritis, and atherosclerosis. In the context of cancer, the rapid and uncontrolled growth and division of cells increase the demand for nutrients and oxygen. Consequently, cancer cells tend to induce angiogenesis to facilitate their growth, dissemination, and invasion of other tissues [1]. This angiogenesis is stimulated by proangiogenic factors produced by the cancer cells themselves and/or cancer-associated stromal cells, thereby enabling cancer invasion and metastasis [2]. This process results in the formation of leaky and immature blood capillaries, which contribute to disease progression [3]. Earlier studies have also shown that in cancer cells angiogenesis is often activated through the epidermal growth factor receptor (EGFR) signaling pathway [1]. Therefore, interrupting this pathway has emerged as a potential target in cancer treatment [4]. In this context, it is essential to highlight that several antiangiogenic drugs, such as axitinib and lenvatinib, are presently used in managing different types of human cancers. These inhibitors operate through different mechanisms, including the blockade of the vascular endothelial growth factor (VEGF) and its receptor. As a result, the formation of new blood capillaries is hindered, preventing the supply of oxygen and nutrients to tumor cells, which ultimately leads to tumor deterioration [5–7]. It is essential to emphasize, however, that the outcome of antiangiogenic drug monotherapy on cancer cells is limited, primarily because they inhibit angiogenesis rather than directly destroying tumor cells. Therefore, antiangiogenic drugs are increasingly being considered as promising agents in combination cancer therapy [8, 9].

Neratinib, a tyrosine kinase inhibitor (TKI) (pan-human epidermal growth factor receptor [HER] inhibitor), targets members of the EGFR family (EGFR, HER2, and HER4) by binding to their intracellular domain. This binding effectively blocks their kinase activity, diminishing their downstream targets [10]. The Food and Drug Administration (FDA) has recently approved neratinib for use as an adjuvant treatment in HER2-positive breast cancer patients who have previously received trastuzumab-based adjuvant therapy [11]. In addition, it is currently being investigated in various clinical trials as a potential treatment for other types of cancer, including non-small cell lung cancer, colorectal cancer, and glioblastoma [9]. However, the effects of neratinib on angiogenesis and the early stages of embryogenesis have not been investigated yet. In this study, we sought to explore the impact of neratinib on angiogenesis using the chorioallantoic membrane (CAM) of chicken embryos as a pre-clinical model. We also investigated the safety of neratinib during the early stages of embryogenesis using the chicken embryo, a model known for its sensitivity in toxicity studies, as reported in several recent investigations [12–16]. Our findings indicate that neratinib treatment can inhibit angiogenesis dose-dependently, positioning it as a potential antiangiogenic candidate in cancer management. The antiangiogenic effects of neratinib, as confirmed by the quantitative real-time polymerase chain reaction (qPCR), are likely mediated through the inhibition of VEGF. Additionally, neratinib exhibits tolerable toxicity during the early stages of normal development. In contrast, on the molecular level, neratinib shows a significant downregulation of several key apoptotic genes when administrated in high doses. However, further studies are warranted to fully understand the mechanisms of action through which neratinib inhibits angiogenesis in the CAM model.

## Materials and methods

### Drugs and reagents

Neratinib (#18404, Cayman Chemical) was initially dissolved in dimethyl sulfoxide (DMSO) to prepare a stock solution with a concentration of 6 mg/mL. To create a working solution, this stock solution was further diluted in DMSO, achieving a final concentration of 0.1 mg/mL.

### Chicken embryos

Fertilized White Leghorn chicken eggs (Arab Qatari for Poultry Production, Qatar) were incubated at 37 ^∘^C with 60% humidity in the MultiQuip incubator. To prevent adhesion between the embryo and its membranes, the eggs were rotated hourly [17]. In this study, embryos at an age of three and five days of incubation were used for the embryogenesis and angiogenesis analysis, respectively. The embryos were treated with varying doses of neratinib (0, 50, 100, and 200 nM), following protocols established by our research group [12–16], and their responses were compared with control embryos treated solely with DMSO. For the angiogenesis analysis, images were taken 48 h post-treatment and subsequently analyzed using the AngioTool Software version 0.6a, as described by Zudaire et al. [18]. For the embryogenesis experiment, embryos were autopsied five days after treatment, with brain, heart, and lung tissues being collected for RNA extraction and subsequent qPCR analysis.

### RNA extraction and qPCR

Total RNA was extracted from tissues (brain, heart, and lung) collected from chicken embryos using the NucleoSpin TriPrep Mini kit (MACHEREY-NAGEL, Germany), following the manufacturer’s instructions. The concentration of the RNA was measured using a nanodrop reader (Thermo-Fisher Scientific, USA), and its purity was evaluated based on a 260/280 nm absorbance ratio. An absorbance ratio of approximately two was indicative of pure RNA. Subsequently, complementary DNA (cDNA) was synthesized from the RNA using the SuperScript™ III First-Strand Synthesis SuperMix kit (Thermo Fisher, USA), adhering to the manufacturer’s protocol. This was followed by the qPCR utilizing the iTaq™ Universal SYBR^®^ Green Supermix kit (BIO-RAD, Australia), again in accordance to the manufacturer’s protocol. The qPCR aimed to investigate the changes of expression in several key genes associated with apoptosis and angiogenesis, including caspase-3, caspase-8, caspase-9, B-cell lymphoma 2 (*Bcl2*) gene, and *Vegf*. The expression of these target genes was normalized to the glyceraldehyde 3-phosphate dehydrogenase (*GAPDH*) gene. The qPCR assay was subsequently performed using the QuantStudio^®^ 5 Real-Time PCR System.

### Cell culture

The impact of neratinib treatment was studied on embryonic fibroblasts (EFBs) and human umbilical vein endothelial cells (HUVEC) (ATCC, USA). EFBs were developed in our laboratory using a 10-day-old chicken embryo. Briefly, to do this, the embryos were first extracted from their eggs. The limbs and internal organs were then excised, and the remaining tissues were subjected to multiple treatments with trypsin-ethylenediaminetetraacetic acid (EDTA) (0.25%) and phenol red (Gibco, Life Technologies). Both cell lines were cultured and grown in complete cell culture media, specifically Gibco^®^ Roswell Park Memorial Institute-1640 (RPMI-1640) media (Gibco, Life Technologies), supplemented with 10% fetal bovine serum (FBS) (Invitrogen, Life Technologies) and 1% PenStrep antibiotic (Invitrogen, Life Technologies) at a temperature of 37 ^∘^C in an atmosphere containing 5% CO_2_. All the experiments were conducted when the cells reached approximately 70%–80% confluence.

### Cell viability assay

EFBs and HUVEC were seeded in 96-well plates (Thermo Fisher Scientific, USA), at a density of 10,000 cells/well, and left to adhere overnight. Subsequently, the cells were treated with varying neratinib concentrations (0, 50, 100, and 200 nM) for 48 h, and compared to untreated controls. AlamarBlue™ Cell viability reagent (Invitrogen, Thermo Fisher Scientific, USA) was used to assess cell viability, following the manufacturer’s protocol. The fluorescence values were recorded at an excitation wavelength of 560 nm and an emission wavelength of 600 nm using an Infinite m200 PRO fluorescent microplate reader (TECAN, Switzerland).

### Ethical statement

The Institutional Bio-Safety Committee of Qatar University approved all chicken embryo experiments (QU-IBC-2019/032-REN2).

### Statistical analysis

The raw data were analyzed using Microsoft Excel and SPSS^®^ 28 software (SPSS Inc., USA). To determine the differences between the treated groups and the control group, a one-way analysis of variance (ANOVA) was conducted, followed by Tukey’s post-hoc test for multiple comparisons. Results were deemed statistically significant at a *P* value of less than 0.05.

## Results

**Figure 1.**
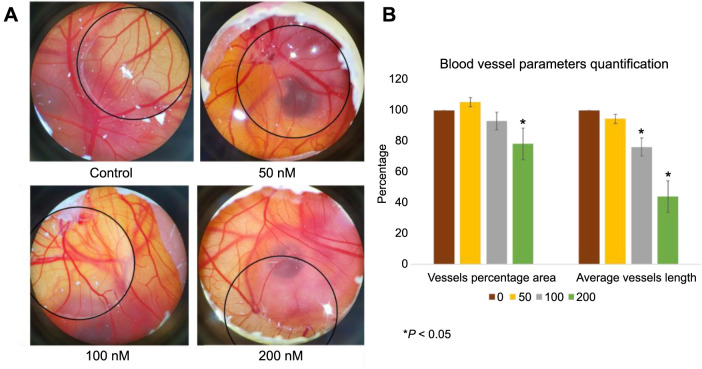
**Angiogenesis assay of the CAM in chicken embryos**. The embryos were treated with 0, 50, 100, and 200 nM of neratinib. (A) Stereomicroscopic images displaying the effects of neratinib treatment on angiogenesis in the CAM. Neratinib treatment inhibited angiogenesis in the CAM at doses of 100 and 200 nM, compared to both the DMSO control and the untreated CAM (*P* < 0.05). The images were taken using a stereomicroscope, 48 h post-treatment (*n* ═ 3). (B) Presenting the quantification of vessel percentage area and average vessel length following neratinib treatment. Neratinib treatment downregulated the blood vessel parameters in the CAM of chicken embryos. The data are presented as mean ± SEM (*n* ═ 3). Statistical analysis was performed using a one-way ANOVA, with Tukey’s post-hoc test conducted for group comparisons. The results were considered statistically significant at *P < 0.05. CAM: Chorioallantoic membrane; DMSO: Dimethyl sulfoxide; SEM: Standard error of the mean; ANOVA: Analysis of variance.

In our study, 5-day-old embryos were treated with different doses of neratinib (0, 50, 100, and 200 nM) for 48 h. As outlined in our previous publications [12–16], the treatment method involved placing a round coverslip directly over the CAM. This setup was crucial for facilitating direct comparisons between areas of the embryo exposed to the treatment and those that were not. Images of the treated and untreated embryos indicated that neratinib inhibited angiogenesis in the exposed areas at concentrations of 100 and 200 nM compared to the unexposed areas and the control group (Figure 1A). In contrast, control embryos exhibited the formation of fine, small capillaries in both DMSO-exposed and DMSO-unexposed areas, an observation not evident in embryos treated with 100 and 200 nM of neratinib (Figure 1). No noticeable effect on angiogenesis was observed visually in embryos treated with 50 nM of neratinib (Figure 1A). Further analysis using AngioTool software to quantify blood vessel parameters revealed that neratinib significantly reduced the vessel percentage area at a dose of 200 nM by approximately 20% compared with the DMSO control. Additionally, treatment with 100 and 200 nM of neratinib resulted in a reduction in the average vessel length in the CAM by about 20% and 65%, respectively, compared to the control group (*P* < 0.05) (Figure 1B).

**Figure 2.**
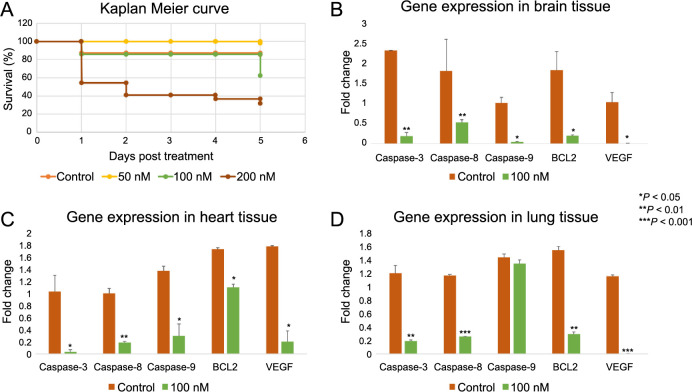
**Survival rate of chicken embryos treated with 0, 50, 100, and 200 nM of neratinib.** (A) Kaplan–Meier survival curve comparing the survival rates of chicken embryos treated with different concentrations of neratinib (0, 50, 100, and 200 nM) over a five-day period post-treatment. A dose-dependent viability is observed. (B–D) qPCR analysis displaying the changes in gene expressions of apoptotic and angiogenic markers in tissues derived from the treated/untreated chicken embryos, namely brain (B), heart (C), and lung (D) tissues. Neratinib treatment led to a significant downregulation of genes associated with apoptosis and VEGF compared to the controls. The data are presented as a percentage of the treatment effect relative to the control, with the values expressed as mean ± SEM (*n* ═ 3). Statistical analysis was performed using a one-way ANOVA, with Tukey’s post-hoc test conducted for group comparisons. The results were considered statistically significant at *P < 0.05 compared to the control. qPCR: Quantitative real-time polymerase chain reaction; VEGF: Vascular endothelial growth factor; SEM: Standard error of the mean; ANOVA: Analysis of variance; BCL2: B-cell lymphoma 2.

Further, we utilized the 3-day-old chicken embryo model to evaluate the embryotoxicity of neratinib after five days of exposure to 0, 50, 100, and 200 nM of neratinib. Our results showed that the toxicity of neratinib becomes significantly pronounced at a dose of 200 nM, in contrast to its lower doses (50 and 100 nM), compared to the control (*P* < 0.001) (Figure 2A). Notably, a significant drop in embryonic survival was observed at a dose of 100 nM on the fifth day of exposure. This data indicated that at doses of 50 nM and 100 nM, neratinib is relatively well-tolerated in the early stages of normal development before five days of exposure. Subsequently, we collected major organs (brain, heart, and lung) from both the treated and untreated chicken embryos for qPCR analysis. This analysis focused on key genes related to apoptosis and angiogenesis, including caspase-3, caspase-8, caspase-9, *Bcl2*, and *Vegf*. The findings revealed that neratinib significantly downregulated the expression of the apoptosis-related genes in the examined organs compared to untreated controls. It is crucial to highlight that while microscopic-level embryonic toxicity was not evident, neratinib did cause deregulation of apoptotic genes on a molecular level. Furthermore, treatment with neratinib resulted in a notable reduction in *Vegf* gene expression across all examined tissues, confirming neratinib’s significant effect at the molecular level (Figure 2B–2D).

**Figure 3.**
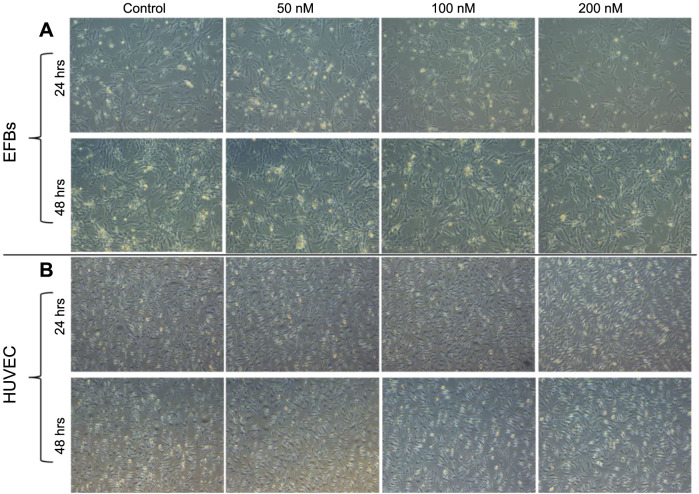
**The effect of neratinib on the cell morphology of EFBs (A) and HUVEC (B)**. The images show no significant morphological changes in either cell line following neratinib treatment across the various concentrations (0, 50, 100, and 200 nM), indicating its safety on these two cell lines. Images were taken at a magnification of 10× following 24 and 48 h of treatment (*n* ═ 3). EFBs: Embryonic fibroblasts; HUVEC: Human umbilical vein endothelial cells.

We further investigated the effects of neratinib on EFBs and HUVEC, using the same doses (0, 50, 100, and 200 nM) for periods of 24 and 48 h. The resulting images indicated that cells treated with neratinib did not exhibit significant morphological changes when compared to the control in both cell lines (Figure 3A and 3B). Nevertheless, a noticeable reduction in cell count was observed at the 200 nM dose, aligning with our in-ovo findings, while lower doses did not show such a decrease (Figure 3).

**Figure 4.**
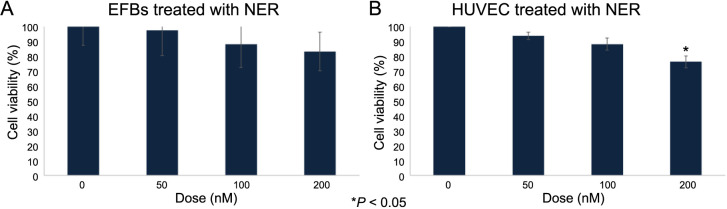
**Effect of neratinib treatment (0, 50, 100, and 200 nM) on EFBs (A) and HUVEC (B) cell viability after 48 h.** A dose-dependent reduction of cell viability was observed only in HUVEC cells. The data are presented as a percentage of the treatment effect relative to the control, with the values expressed as mean ± SEM (*n* ═ 3). Statistical analysis was performed using a one-way ANOVA, with Tukey’s post-hoc test conducted for group comparisons. The results were considered statistically significant at *P < 0.05 compared to the control. EFBs: Embryonic fibroblasts; HUVEC: Human umbilical vein endothelial cells; SEM: Standard error of the mean; ANOVA: Analysis of variance; NER: Neratinib.

After assessing the viability of EFBs and HUVEC cells following neratinib treatment, we noted a similar trend, with no significant decrease in cell viability (Figure 4A and 4B). The only exception was in HUVEC cells at the 200 nM neratinib concentration, where a significant decrease in viability was noted (*P* < 0.05) (Figure 4B).

## Discussion

Antiangiogenic drugs are gaining prominence in the treatment of various pathological conditions, particularly cancer [2]. Disrupting the pre-existing blood vessels and preventing the formation of new blood capillaries is the rationale behind using antiangiogenic drugs in cancer treatment [19, 20]. Subsequently, this will deprive tumor cells of nutrients and oxygen and will result in cancer regression [9]. The VEGF receptor (VEGFR) family is recognized as a primary angiogenesis regulator [21, 22], which makes it an essential target for antiangiogenic drugs [23, 24]. For instance, bevacizumab is a VEGF-A monoclonal antibody that binds to VEGF, blocking its interaction with its receptor [25, 26]. Bevacizumab, along with other antiangiogenic agents, such as sorafenib, sunitinib, and pazopanib, has shown good clinical efficacy in several tumor types [26, 27]. However, the development of primary and acquired resistance results in the failure of antiangiogenic treatment in certain cancers [28–30], underscoring the importance of developing new drugs that target angiogenesis for improved cancer management. This study reports a novel antiangiogenic role of neratinib, a TKI (pan-HER inhibitor), approved for treating HER2-positive breast cancer [10]. Neratinib works by inhibiting EGFR phosphorylation, subsequently blocking its downstream signaling. Our findings showed that neratinib inhibits angiogenesis in the CAM of chicken embryos in a dose-dependent manner compared to the controls (Figure 1). In this regard, it has been previously reported that cancer cells can indirectly activate neovascularization by triggering the EGFR signaling pathway and producing proangiogenic factors [1, 31]. Consequently, blocking the EGFR has been reported to inhibit angiogenesis in renal cancer in vivo [32].

Furthermore, activation of the EGFR pathway has been shown to contribute to the development of resistance to antiangiogenic treatments [28]. Other TKIs, such as sorafenib, exhibit antiangiogenic effects that are mediated through blocking the EGFR signaling, which corroborates our findings [33]. Moreover, various studies have suggested that a dual strategy, targeting both VEGFR and EGFR by combination therapy, may be a promising approach in cancer treatment [34, 35]. For instance, combining bevacizumab with EGFR blockers, such as neratinib, might enhance the clinical outcomes in cancer management. However, the effect of neratinib on VEGF members and their receptors has not yet been investigated, to the best of our knowledge. Hence, we herein report for the first time that neratinib treatment significantly downregulates VEGF expression, which might be the primary mediator in the observed antiangiogenic effects of neratinib (Figure 2B–2D). Conversely, our data reveal that a significant reduction in HUVEC cell viability was only noted at a neratinib concentration of 200 nM in vitro (Figure 4B). This suggests that neratinib’s inhibition of angiogenesis may result from modulating signaling pathways within endothelial cells, rather than merely reducing cell viability.

It is important to highlight that chicken embryos represent a robust in-ovo model for studying angiogenesis and drug toxicity. This model is simple, fast, and cost-effective [36–38]. However, our data need validation using alternative models, preferably an in-vivo model, to fully elucidate neratinib’s impact on angiogenesis and to address evolutionary differences between species. Exploring the toxicity of neratinib in the early stages of embryogenesis provides critical information regarding the potential drug administration risks during pregnancy [39]. It also sheds light on the mechanisms underlying neratinib’s toxic effects, which are consistent with those observed with other multi-kinase inhibitors that are known to exhibit undesired effects [40, 41]. Our data indicate that using neratinib at doses of 50 and 100 nM for a short time is not toxic during embryogenesis. However, on the fifth day of exposure, a dose of 100 nM of neratinib reduces embryonic viability to approximately 60%. Additionally, embryonic toxicity is highly pronounced at a higher dose of neratinib (200 nM), resulting in approximately 70% mortality among the exposed embryos (*P* < 0.05) (Figure 2A). These findings were confirmed by qPCR, which suggested that long-term exposure to 100 nM of neratinib (five days) downregulates key genes responsible for apoptosis, namely caspase-3, caspase-8, caspase-9, and *Bcl2*. Next, we used primary EFBs to confirm our findings in vitro. The obtained results corresponded to what was found in-ovo, as the greatest reduction in cell viability was observed upon treatment with 200 nM of neratinib (approximately 80%), although it was not statistically significant (*P* > 0.05) (Figure 4A). In this context, other TKIs were found to induce toxicity in zebrafish embryos in a dose-dependent manner. More specifically, EGFR inhibitors gefitinib and afatinib led to hepatotoxicity in zebrafish larvae, mainly by disrupting the expression of several genes related to apoptosis, such as *Bcl2*, BCL-2 associated X protein (*Bax*) gene, caspase-3, caspase-9, and caspase-8 [42], similar to what is found in our study. Furthermore, lenvatinib, a multi-kinase inhibitor, induced cardiotoxicity mediated by upregulating apoptosis proteins in zebrafish embryos [43]. The same pattern was observed for imatinib and sunitinib, where both exhibit high toxicity during pregnancy [44, 45]. Collectively, neratinib appears to exhibit a safer profile compared to other TKIs. Nonetheless, it is necessary to highlight the importance of conducting intensive safety studies before considering the administration of TKIs during pregnancy. Altogether, our data highlight a new potential use of neratinib as an antiangiogenic agent in cancer management, with a relatively safe profile during the early stages of embryogenesis.

## Conclusion

We propose a novel potential application of neratinib, a pan-HER inhibitor, as an antiangiogenic agent. Further research is warranted to fully understand the interactions between neratinib and VEGFRs and to clarify its mechanism of action. Our study indicates that neratinib combined with anti-VEGF drugs could be a promising candidate in cancer management, especially HER2-positive tumors. Moreover, neratinib has exhibited an acceptable safety profile at the early stages of embryogenesis. Nonetheless, further investigations are essential to confirm the safety of neratinib during pregnancy using other pre-clinical models.
